# Types of Carbohydrates Intake during Pregnancy and Frequency of a Small for Gestational Age Newborn: A Case-Control Study

**DOI:** 10.3390/nu11030523

**Published:** 2019-02-28

**Authors:** Carmen Amezcua-Prieto, Juan Miguel Martínez-Galiano, Naomi Cano-Ibáñez, Rocío Olmedo-Requena, Aurora Bueno-Cavanillas, Miguel Delgado-Rodríguez

**Affiliations:** 1 Department of Preventive Medicine and Public Health, University of Granada, 18071 Granada, Spain; carmezcua@ugr.es (C.A.-P.); ncaiba@ugr.es (N.C.-I); rocioolmedo@ugr.es (R.O.-R); abueno@ugr.es (A.B.-C.); 2 Consortium for Biomedical Research in Epidemiology and Public Health (CIBERESP), 28029 Madrid, Spain; juanmimartinezg@hotmail.com (J.M.M.-G.); mdelgado@ujaen.es (M.D.-R.); 3 Instituto de Investigación Biosanitaria ibs.GRANADA, Complejo Hospitales Universitarios de Granada/Universidad de Granada, 18071 Granada, Spain; 4 Department of Nursing, University of Jaén, Campus de Las Lagunillas s/n, Building B3 Office 266, 23071 Jaén, Spain; 5 Division of Preventive Medicine and Public Health, University of Jaen, 23071 Jaen, Spain

**Keywords:** carbohydrates, pregnancy, refined carbohydrates, complex carbohydrates, small for gestational age

## Abstract

The objective of this study was to assess the relationship between consumption of different types of carbohydrates (CHO) during pregnancy and the risk of having a small for gestational age (SGA) newborn. A retrospective matched case–control design was carried out with a total of 518 mother-offspring pairs. A total of 137 validated items were included in the food frequency questionnaire (FFQ). Conditional logistic regression models were used to calculate crude odds ratios (cORs) and adjusted odds ratios (aORs) with 95% confidence intervals (CIs). Having more than 75 g/day of brown bread showed an inverse association with SGA (aOR = 0.64, CI 0.43–0.96). In contrast, an intake of industrial sweets more than once a day (aOR = 2.70, CI 1.42–5.13), or even 2–6 times a week (aOR = 1.84, CI 1.20–2.82), increased the odds of having a SGA newborn. During pregnancy, the higher the increase of wholegrain cereal and bread, the lower the possibility of having a SGA newborn, but the opposite occurred with refined sugar products—just consuming industrial bakery products or pastries twice a week increased the odds of having an SGA infant. Case–control studies cannot verify causality and only show associations, which may reflect residual confusion due to the presence of unknown factors. It is possible that a high consumption of sugary foods is a marker of a generally poor lifestyle.

## 1. Introduction

Around 19% of newborns from low- and middle-income countries are small for gestational age (SGA)—smaller in size than normal for their gestational age—with weight below the 10th percentile for the gestational age [1]. SGA infants face an increased risk of experiencing later morbidity in childhood, poor linear growth, and chronic non-communicable disease in adulthood [2]. 

A well-balanced and healthy maternal diet during pregnancy seems to reduce the likelihood of having an SGA newborn [3,4,5]. According to the European Food Safety Authority Scientific Committee [6], carbohydrates are basic components in the maternal diet and should represent between 45% and 60% of the calories in a healthy diet in the adult population as well as during pregnancy. The Spanish Society of Community Nutrition [7] suggests an intake of 4–5 portions of carbohydrates per day during pregnancy.

There are different types of carbohydrates comprising sugars, starchy carbohydrates and dietary fiber [8]. In general, starchy carbohydrates (pasta, potatoes, bread, cereals and rice), mainly whole grain cereals and complex fiber-rich carbohydrates, are the core of a healthy diet [7,9]. Refined sugar, such as sugar-sweetened carbonated soft drinks (SSC) or foods containing refined sugar, reduces the total vitamin and mineral density of the diet by displacing more nutrient-dense foods [10]. In fact, some randomized controlled trials pointed out that pregnant women with a lower glycemic index (LGI) diet had higher micronutrient intake, and therefore better newborn weight [11] and less probability of having a newborn with macrosomia [12]. On the other hand, a higher intake of refined grains and added sugars during pregnancy increased the risk of gestational diabetes mellitus [13], reduced birthweight, and augmented the risk of an SGA infant [14,15]. 

Observational studies have shown a relationship between the energy intake of carbohydrates (CHO) and offspring outcomes, but they were not focused on CHO types [16,17,18,19]. For instance, the Healthy Start Study*,* in USA found that regardless of pre-pregnancy body mass index, the maternal total CHO intake during pregnancy was associated with a 2–9 g increase in neonatal fat mass, in a prospective pre-birth cohort of 1040 mother-offspring pairs [16,18]. Sharma et al. observed that an additional 10 g/day CHO consumption was associated with an increase of 4 g in birth weight [19]. Moreover, it has been observed in a cohort of 2035 births from an urban Indian population that the risk of having a SGA newborn increased with higher CHO intake in the first trimester (aOR per 5% of energy: 1.15; CI, 1.01–1.32), specifically in male births [17]. Nevertheless, the relationship between maternal intake of different types of CHO and having an SGA newborn remains unclear. A healthy diet that incorporates more wholegrain products instead of unhealthy, refined sugar foods may act as a protective factor against having an SGA newborn. 

The present study aimed to determine the associations between intake of various CHOs during pregnancy and the odds of having an SGA newborn.

## 2. Materials and Methods

The study population included women that attended five hospitals of Eastern Andalusia (Spain): The University of Jaen Hospital (UJH), Ubeda Hospital (UB), the University of Granada Hospitals (two centers) (UGH), and Poniente Hospital (PH). Taken together, these hospitals serve 1.8 million people. The SGA and control groups were recruited from May 15, 2012 through July 15, 2015. The Ethics Committees of the hospitals authorized this study. All women included in this study provided informed consent. We estimated the appropriate sample size based on the results of a similar study [20]. To detect a significant (*p* < 0.05) OR of 0.6 between extreme quintiles with a statistical power of 80%, we estimated that 447 pairs of cases and controls were required.

### 2.1. SGA Group

**Figure 1:**
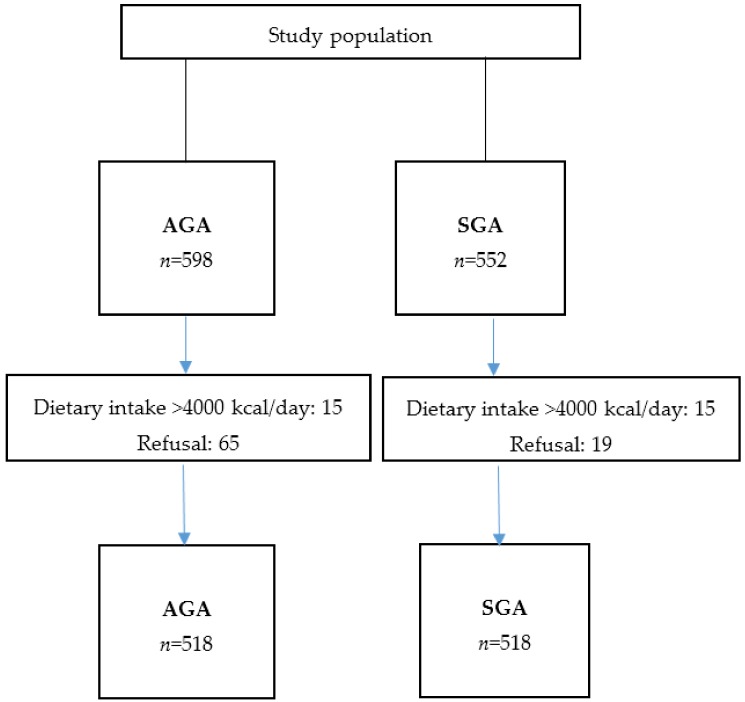
Study population flow chart.

Tables previously developed for the Spanish population [21] classified SGA newborns as infants with birthweights below the 10th percentile of infants at the same gestational age. Mothers were eligible for the SGA group when they had delivered a single live SGA newborn with no congenital malformation during the study period; they had to reside in the referral area of the hospital; and they had to have an adequate understanding of the Spanish language. The control group comprised women that gave birth to babies adequate for gestational age (AGA). Mothers who gave birth to babies large for gestational age (LGA) were excluded. Nineteen women declined participation. A total of 533 women were included in the SGA case group from the following hospitals: UJH (*n* = 79), UGH (*n* = 369), UH (*n* = 46), and PH (*n* = 39) (Figure 1).

### 2.2. Control Group

Within a week after including each woman in the SGA group, women of similar age at delivery (± 2 years) were selected at the same hospital for the control group. Women eligible for the control group delivered a non-SGA newborn (birthweight above the 10th percentile), but otherwise had to meet the same inclusion criteria required for the SGA group (residence in the referral area of the hospital, single pregnancy with no malformation, and an adequate understanding of Spanish). Sixty-five women declined participation.

### 2.3. Data Collection

We used three sources of data: Midwives conducted first, personal interviews within two days after delivery; second, we reviewed clinical charts; and finally, we reviewed prenatal care records. 

We collected the following data: Mother’s vital data, including age at pregnancy, race, education level, marital status, socioeconomic class, and occupation; obstetric history, including parity and abortions, previous adverse perinatal outcomes, and conditions during pregnancy (infections, preeclampsia, diabetes, and other obstetric conditions); birthweight, measured in grams, in the delivery room; drugs or medications, including prescribed drugs and over-the-counter medications; social class, which included five main levels, ranging from I (the highest) to V (the lowest), according to the Spanish Society of Epidemiology classification system [22]; the Kessner index, a measure of prenatal care utilization (number of visits and date of first visit) [23]; and toxic habits, including alcohol consumption during and before pregnancy (assessed with a structured questionnaire, which queried the number and type of drinks taken on weekdays, weekends, including Friday evening, and holidays, including holiday eves); and smoking during pregnancy. The same data collection procedures were performed for SGA and control groups.

### 2.4. Dietary Assessment

The midwives provided the food frequency questionnaire (FFQ) to women after birth. The completion and return of the questionnaire confirmed participation in the study. The baseline questionnaire included a 137-item FFQ, previously validated in Spain, with open questions about the use of dietary supplements [24,25]. The questions were based on typical portion sizes, and 9 options described the frequency of intake in the previous year for each food item (range: Never or almost never to ≥6 times a day). For each FFQ food item, we estimated (1) the average amount of food consumed (grams) multiplied by the intake frequency; (2) the average total energy intake; and (3) the average intake of macro and micronutrients, based on the latest available information from Spanish food composition tables [26,27]. Additionally, we asked each woman whether she had modified her intake of any items on the FFQ due to her pregnancy (i.e., lower, higher, or unchanged intake).

Bakery and sweets were included in the FFQ, as follows: Simple biscuits, whole biscuits, chocolate biscuits, home bakery, croissant, pastries, doughnut, muffins, cakes, churros, chocolates, cocoa, nougat, shortbreads; and cereals: White bread, sliced bread, cereals in breakfast, whole cereals, white rice, pasta, and pizza.

After computing the total energy intake, we excluded 15 matched pairs, due to unreliable dietary assessments (the total energy calculations indicated intakes above 4000 kcal/day). Thus, 518 matched SGA and control pairs were included in the final analysis.

### 2.5. Statistical Analysis

Quantitative variables are expressed as the mean, standard deviation (SD), and range, and frequencies are expressed in absolute and relative terms. The intake frequencies were derived from the FFQ; each respondent indicated intakes for the different types of carbohydrates such as: never; 1–3 servings/month; 1 serving/week; or >1 serving/week). 

We used the residuals method to adjust food and nutrient intakes to the total energy intake, for the SGA and control groups in separates ways, as recommended previously [28]. Nutrient intakes were stratified into quintiles, according to intakes observed in controls (which represent the general population); thus, the quintile was used to stratify the total amount of cereals, wholegrain cereal and bread and white cereals plus rice and pasta in the control group were applied to intakes reported in the SGA group. 

We implemented conditional logistic regression models to calculate crude odds ratios (cORs) and adjusted odds ratios (aORs) with their 95% CIs. The lowest quintile (Q1) was taken as the reference, and the main comparison was to the highest quintile (Q5). Trend analyses were performed with quintiles in logistic regression models, where the median intake for each quintile was introduced into the model. To control for confounding variables, intermediate variables were discarded and only variables that changed the diet coefficient by more than 10% were retained in the logistic models. The model was adjusted for energy intake, maternal educational level, smoking, pre-gestational BMI, parity and a previous preterm or low birthweight newborn. All *p*-values are 2-tailed. Statistical significance was set at *p* < 0.05. We performed all analyses with Stata 14 (College Station, TX, USA).

## 3. Results

In Table 1 we can appreciate the main characteristics of cases and controls pregnant women. Cases were less frequently married, they had more previous preterm or low-birthweight newborns, they smoked more than controls, and the frequency of preeclampsia and intrauterine growth restriction was also higher in cases than in controls women.

Table 2 shows the frequency of intake of different types of bread and cereals in cases and controls women and the association with SGA newborns. Having brown bread more than once a day (>75 g/day) had a protective effect against SGA aOR = 0.64 (0.43–0.96). In contrast, the consumption of white bread more than twice a day (>150 g/day) aOR = 1.19 (0.78–1.81) or pizza (>200 g) one or more times a week aOR = 1.14 (0.87–1.48) were positively but slightly associated with having an SGA, with no statistically significant results.

The daily intake (g/day) of cereals from cases and controls and their association with SGA newborns appears in Table 3. The total amount of wholegrain cereal and bread, in grams per day, was higher for controls than cases. The higher the increase of wholegrain cereal and bread, the lower the chance to have an SGA newborn. The most beneficial effects were observed for consumption between 10.57 and 45.61 g/day. The consumption of more than 45.61 grams per day (Q5) showed an inverse association with the outcome aOR = 0.59 (0.39–0.91). Total amount of white cereals, rice and pasta was not associated with having an SGA newborn (Table 3).

Table 4 shows the frequency of intake of different types of sweets and the association with SGA. Refined sugars, such as industrial bakery and pastries more than twice a week (≥100 g), compared with not consuming them, were associated with SGA aOR = 1.59 (1.02–2.48). Having more than two doughnuts a week compared with non-consuming increased the odds of having an SGA newborn: aOR = 2.10 (1.02–4.29). A dose–response relationship was observed for the combination of industrial sweets (bakery, pastries, doughnuts and muffins) and the study outcome. A combination of industrial sweets resulting in consumption more than 2–6 times a week increased the odds of an SGA newborn: aOR = 1.84 (1.20–2.82). The association was higher with an intake of industrial sweets more than once a day: aOR = 2.70 (1.42–5.13). During pregnancy, 12.4% of SGA mothers increased intake (14.9% of AGA mothers), whereas 17.6% reduced their consumption (18.7% of AGA mothers), this difference being non-significant (*p* = 0.387).

## 4. Discussion

The total consumption of wholegrain cereal and bread in grams per day was higher in control women. The intake of wholegrain bread during pregnancy had a protective effect against SGA in our sample of 518 cases and 518 controls. On the contrary, refined sugars and the intake of factory made sweets increased the odds of having an SGA newborn.

### Strengths and Limitations

Some strengths in our study were the following: A small number of women refused to participate when selection took place—only 3% in the SGA group and 11% in the control group. Consequently, the selection bias was rather low. To reduce information bias, the 137-item FFQ was previously validated in the Spanish population [24,25]. This is a self-administered tool supervised by midwives. Since there are no recommendations on specific carbohydrate intake for pregnancy, we believe that the answers given by cases and controls do not differ, and a non-differential misclassification bias is assumed. The control group was density matched to the SGA group, decreasing the seasonal influence on carbohydrates intake between groups. The matching for age avoids the preference of carbohydrates by young or older pregnant women. We minimized biases, adjusting by the best known risk factors of SGA in the multivariable analyses such as maternal education level, pre-pregnancy body mass index, smoking, parity, and previous preterm or low birthweight newborn.

There are some drawbacks to the study: Case–control studies cannot verify causality, only associations, which may reflect residual confusion due to the presence of unknown factors. Nevertheless, information on the most important risk factors was collected. Notwithstanding, it may be possible that underreporting of items that are harmful for health, such as industrial bakery, pastries and doughnuts, be present. As it has been mentioned in the results, there were not differences in reporting changes of intake during pregnancy between cases and controls. 

The association between total carbohydrates intake during pregnancy and SGA or body composition has been previously studied. In an observational cohort study of 2035 births from an urban Indian population [17], Mukhopadhyay et al. reported an increased risk of SGA with higher intakes of CHO (aOR per 5% of energy from carbohydrate: 1.15 (CI, 1.01–1.32)). According to this, the same authors observed that dietary intake of >70% of energy from CHO was associated with an increased risk of SGA in male births (aOR: 1.67; 95% CI: 1.00–2.78) [17]. On the contrary, in the Healthy Start Study on pregnant women, CHO intake was related to a 2–9 g (*p* = 0.02) increase in neonatal fat mass, independent of pre-pregnancy BMI [16], and in the GUSTO Study in Singapore, it was observed that a lower CHO diet during pregnancy is associated with lower abdominal internal adipose tissue (IAT) in the neonates (beta: −0.18 mL (CI: −0.35, −0.001 mL)), being stronger the association in boys than in girls (*p*-value < 0.05) [29]. 

There is evidence that an increased diet quality is linearly associated with a reduced likelihood of SGA (*p* for trend = 0.03); this was observed among 862 women enrolled in the New Hampshire Birth Cohort study [4]. The same tendency was observed in our sample when pregnant women have a good adherence to the Mediterranean diet pattern [5], and specifically we have found that the total amount of wholegrain cereal and bread in g/day (mainly categories Q4 and Q5) is negatively associated with SGA. In a previous case control study of 844 cases (SGA) and 870 controls (AGA, appropriate size for gestational age), carbohydrate-rich foods (potatoes, rice, noodles, pasta, bread and breakfast cereals) were also associated (*p* = 0.04) with a reduced risk of SGA [3].

The association between specific types of carbohydrates consumed during pregnancy, such as rich food carbohydrates, wholegrain cereals, and industrial bakery, and the probability of having a SGA has been less studied. The influence of maternal macronutrient dietary glycemic index (GI) on neonatal body composition could have an effect on neonatal growth and body composition. In late pregnancy, higher dietary GI has been associated with lower fat free mass index (*p* = 0.01), reducing indices of both lean mass and adiposity [30]. According to the quality of carbohydrates, those with lower glycemic nature improve micronutrient intake, probably because there is a high consumption of nutrient-dense foods such as fruit, vegetables, nuts, dairy products and legumes [11]. This could explain our results relating to the intake of one or more a day of simple carbohydrates and industrial bakery pastries, doughnuts and muffins, and the increasing effect of nearly three times higher chance of having a SGA newborn. 

The effect of sugar CHO intake during pregnancy on birth weight might be explained by the effect of increased glycaemia and insulinemia resulting in elevated blood pressure and decreased placental blood flow and fetal growth [14]. It has been shown that the insulin-antagonizing effects of growth hormone (which is known to be elevated in SGA infants) are main factors involved in fetal growth [31]. Moreover, mean placental and birth weights are inversely related to a mother's macronutrients intake in early pregnancy. Division of carbohydrate into total sugars and starch showed that the relations between mean placental and birth weights with intakes of sugars were stronger than those with consumptions of starch **[32]**.

Finally, around 60% of total diet energy intake during pregnancy must come from carbohydrates [6]. A general recommendation for a healthy diet during pregnancy is an intake of 4–5 portions of carbohydrates per day [7,9]. Pregnant women should select those carbohydrate-rich foods with lower glycemic index, such as wholegrain cereal and bread, and reject refined sugars, as our result pointed out.

## 5. Conclusions

In conclusion, having more than 75 grams per day of whole wheat bread during pregnancy is negatively associated with the odds of having a SGA newborn, whereas consuming more than two simple carbohydrates and industrial bakery pieces per week (such as pastries or doughnuts) increase the odds of having a SGA infant. Moreover, the higher the increase of wholegrain cereal and bread during pregnancy, the lower the possibility of having a SGA newborn, but the opposite occurs with refined sugar products. Diet intervention is necessary to reduce the probability of having an SGA newborn. During pregnancy, women should be informed about the necessity to diminish their intake of refined CHO and to augment the complex fiber-rich CHO consumption during pregnancy.

## Figures and Tables

**Table 1:** Description of the study population.

Variable	Cases *n* (%)	Controls *n* (%)	*p-*Value
Marital status			0.036
Single	37 (7.1)	42 (8.1)	
Stable couple	161 (31.1)	124 (23.9)	
Married	320 (61.8)	352 (68.0)	
Education level			0.084
Primary	112 (21.6)	93 (17.9)	
High school, not ended	42 (8.1)	28 (5.4)	
High school	185 (35.7)	190 (36.7)	
University	179 (34.6)	207 (40.0)	
Parity			0.080
Primiparous women	331 (63.9)	298 (57.5)	
Secundiparous women	147 (28.4)	180 (34.8)	
Multiparous women (≥2 previous children)	40 (7.7)	40 (7.7)	
Previous preterm or low-birthweight newborn	64 (12.4)	26 (5.0)	< 0.001
Kessner index (prenatal care)			0.737
Adequate	259 (50.0)	253 (48.8)	
Intermediate	185 (35.7)	182 (35.2)	
Inadequate	74 (14.3)	83 (16.0)	
Smoking during pregnancy	149 (28.8)	80 (15.4)	< 0.001
Preeclampsia	46 (8.9)	11 (2.1)	< 0.001
Intrauterine growth retardation	141 (27.2)	8 (1.5)	< 0.001
Weight gain during pregnancy (g/week), mean (SD)	278 (121)	310 (114)	< 0.001
Pre-pregnancy body mass index, mean (SD)	23.1 (4.5)	23.9 (4.1)	< 0.001
Alcohol intake (g/week), mean (SD)	4.2 (18.5)	3.1 (15.2)	0.312

**Table 2:** Frequency of intake of different types of bread and cereals and risk of SGA.

Intakes (g) and Frequency	Cases *n* (%)	Controls *n* (%)	cOR (95% CI)	aOR ^1^ (95% CI)
White bread (75 g)				
Never	126 (24.3)	145 (28.0)	1 (ref.)	1 (ref.)
vs. 2+ a day	83 (16.0)	76 (14.7)	1.27 (0.85–1.89)	1.19 (0.78–1.81)
Whole wheat bread (75 g)				
Never	333 (64.3)	278 (53.7)	1 (ref.)	1 (ref.)
vs. 1+ a day	60 (11.6)	80 (15.4)	0.60 (0.41–0.88)	0.64 (0.43–0.96)
Cereals (30 g)				
Never	356 (68.7)	341 (65.8)	1 (ref.)	1 (ref.)
vs. 2+ a week	72 (13.9)	88 (17.0)	0.78 (0.55–1.11)	0.70 (0.48–1.03)
Wholegrain cereals (30 g)				
Never	341 (65.8)	321 (62.0)	1 (ref.)	1 (ref.)
vs. 2+ a week	67 (12.9)	77 (14.9)	0.81 (0.56–1.18)	0.88 (0.59–1.31)
Rice (60 g in raw)				
Never	76 (14.7)	78 (15.1)	1 (ref.)	1 (ref.)
vs. 2+ a week	36 (7.0)	46 (8.9)	0.82 (0.49–1.38)	1.00 (0.57–1.76)
Pasta (60 g in raw)				
≤1–3 a month	50 (9.7)	45 (8.7)	1 (ref.)	1 (ref.)
vs 2+ a week	95 (18.3)	116 (22.4)	0.72 (0.44–1.18)	0.80 (0.47–1.37)
Pizza (1 portion of 200g)				
≤ 1–3 a month	210 (40.5)	230 (44.4)	1 (ref.)	1 (ref.)
vs. 1+ a week	308 (59.5)	288 (55.6)	1.18 (0.92–1.51)	1.14 (0.87–1.48)

^1^ Adjusted for maternal education level, pre-pregnancy body mass index, smoking, parity, and previous preterm / low birthweight newborn; ref.: reference.

**Table 3:** Daily intake (g/day) of cereals and odds of having a small at gestational age (SGA) newborn.

Quintiles Intake (g/day)	Cases *n* (%)	Controls *n* (%)	cOR (95% CI)	aOR ^1^ (95% CI)
Total amount of cereals				
Q1 (≤117.57)	115 (22.2)	104 (20.1)	1 (ref.)	1 (ref.)
Q2 (117.58–154.63)	75 (14.5)	104 (20.1)	0.65 (0.43–0.97)	0.68 (0.44–1.05)
Q3 (154.64–189.96)	99 (19.1)	103 (19.9)	0.89 (0.61–1.31)	0.93 (0.62–1.40)
Q4 (189.97–239.01)	130 (25.1)	104 (20.1)	1.17 (0.79–1.73)	1.14 (0.76–1.73)
Q5 (>239.01)	99 (19.1)	103 (19.9)	0.89 (0.60–1.32)	0.84 (0.55–1.29)
Total amount of wholegrain cereal and bread				
Q1 (≤0.10)	154 (29.7)	104 (20.1)	1 (ref.)	1 (ref.)
Q2 (0.11–2.08)	105 (20.3)	104 (20.1)	0.67 (0.45–0.99)	0.80 (0.53–1.21)
Q3 (2.09–10.56)	90 (17.4)	103 (19.9)	0.56 (0.37–0.83)	0.60 (0.39–0.91)
Q4 (10.57–45.61)	84 (16.2)	104 (20.1)	0.54 (0.37–0.80)	0.54 (0.35–0.81)
Q5 (>45.61)	85 (16.4)	103 (19.9)	0.54 (0.37–0.80)	0.59 (0.39–0.91)
Total amount of white cereals + rice & pasta				
Q1 (≤ 27.65)	117 (22.6)	104 (20.1)	1 (ref.)	1 (ref.)
Q2 (27.66–51.53)	97 (18.7)	104 (20.1)	0.85 (0.58–1.23)	0.86 (0.58–1.30)
Q3 (51.54–79.10)	100 (19.3)	103 (19.9)	0.88 (0.61–1.26)	0.88 (0.60–1.30)
Q4 (79.11–120.79)	93 (18.0)	104 (20.1)	0.80 (0.54–1.19)	0.88 (0.58–1.34)
Q5 (>120.79)	111 (21.4)	103 (19.9)	0.95 (0.65–1.41)	0.95 (0.63–1.43)

^1^ Adjusted for maternal education level, pre-pregnancy body mass index, smoking, parity, and previous preterm / low birthweight newborn; ref.: reference.

**Table 4:** Frequency of intake of different types of sweets and odds of SGA (continuation).

Frequency of Intake	Cases *n* (%)	Controls *n* (%)	cOR (95% CI)	aOR ^1^ (95% CI)
Industriall bakery and pastries (1 unit/50 g)				
Never	158 (30.5)	174 (33.6)	1 (ref.)	1 (ref.)
vs. 2+ a week	74 (14.3)	57 (11.0)	1.44 (0.95–2.17)	1.59 (1.02–2.48)
Doughnuts (1 unit)				
Never	238 (46.0)	263 (50.8)	1 (ref.)	1 (ref.)
vs. 2+ a week	27 (5.2)	12 (2.3)	2.42 (1.21–4.84)	2.10 (1.02–4.29)
Industrial muffins (1–2 units)				
Never	159 (30.7)	196 (37.8)	1 (ref.)	1 (ref.)
vs. 2+ a week	56 (10.8)	43 (8.3)	1.61 (1.03–2.55)	1.63 (0.99–2.66)
Industrial sweets (bakery and pastries, doughnuts, muffins)				
Never	63 (12.4)	92 (18.2)	1 (ref.)	1 (ref.)
1–3 a month	64 (12.6)	66 (13.0)	1.41 (0.85–2.32)	1.59 (0.93–2.71)
1 a week	156 (30.7)	169 (33.4)	1.37 (0.91–2.08)	1.54 (0.99–2.39)
2–6 a week	186 (36.5)	155 (30.6)	1.73 (1.15–2.58)	1.84 (1.20–2.82)
1+ a day	40 (6.9)	24 (4.7)	2.36 (1.29–4.35)	2.70 (1.42–5.13)
*p* for trend			0.002	0.002

^1^ Adjusted for maternal education level, pre-pregnancy body mass index, smoking, parity, and previous preterm/low birthweight newborn; ref.: reference.
